# Soluble epoxide hydrolase derived lipid mediators are elevated in bronchoalveolar lavage fluid from patients with sarcoidosis: a cross-sectional study

**DOI:** 10.1186/s12931-018-0939-0

**Published:** 2018-12-03

**Authors:** Marcus O. D. Sjödin, Antonio Checa, Mingxing Yang, Sven-Erik Dahlén, Åsa M. Wheelock, Anders Eklund, Johan Grunewald, Craig E. Wheelock

**Affiliations:** 1 Division of Physiological Chemistry II, Department of Medical Biochemistry & Biophysics, Karolinska Institutet, 17177 Stockholm, Sweden; 2 Experimental Asthma & Allergy Research, Institute of Environmental Medicine, Karolinska Institutet, Stockholm, Sweden; 3 Respiratory Medicine Unit, Department of Medicine and Center for Molecular Medicine (CMM), Karolinska Hospital and Karolinska Institutet, Stockholm, Sweden

**Keywords:** Sarcoidosis, Eicosanoids, Sphingolipids, Endocannabinoids, Mass spectrometry, Inflammation

## Abstract

**Background:**

Sarcoidosis is a systemic inflammatory multi-organ disease almost always affecting the lungs. The etiology remains unknown, but the hallmark of sarcoidosis is formation of non-caseating epithelioid cells granulomas in involved organs. In Scandinavia, > 30% of sarcoidosis patients have Löfgren’s syndrome (LS), an acute disease onset mostly indicating a favorable prognosis. The impact of dysregulation of lipid mediators, which has been investigated in other inflammatory disorders, is still unknown.

**Methods:**

Using three different liquid chromatography coupled to tandem mass spectrometry targeted platforms (LC-MS/MS), we quantified a broad suite of lipid mediators including eicosanoids, sphingolipids and endocannabinoids in bronchoalveolar lavage (BAL) fluid from pulmonary sarcoidosis patients (*n* = 41) and healthy controls (*n* = 16).

**Results:**

A total of 47 lipid mediators were consistently detected in BAL fluid of patients and controls. After false discovery rate adjustment, two products of the soluble epoxide hydrolase (sEH) enzyme, 11,12-dihydroxyeicosa-5,8,14-trienoic acid (11,12-DiHETrE, *p* = 4.4E-5, q = 1.2E-3, median fold change = 6.0) and its regioisomer 14,15-dihydroxyeicosa-5,8,11-trienoic acid (14,15-DiHETrE, *p* = 3.6E-3, q = 3.2E-2, median fold change = 1.8) increased in patients with sarcoidosis. Additional shifts were observed in sphingolipid metabolism, with a significant increase in palmitic acid-derived sphingomyelin (SM16:0, *p* = 1.3E-3, q = 1.7E-2, median fold change = 1.3). No associations were found between these 3 lipid mediators and LS, whereas levels of SM 16:0 and 11,12-DiHETrE associated with radiological stage (*p* < 0.05), and levels of 14,15-DiHETrE were associated with the BAL fluid CD4/CD8 ratio.

**Conclusions:**

These observed shifts in lipid mediators provide new insights into the pathobiology of sarcoidosis and in particular highlight the sEH pathway to be dysregulated in disease.

**Electronic supplementary material:**

The online version of this article (10.1186/s12931-018-0939-0) contains supplementary material, which is available to authorized users.

## Background

Sarcoidosis is a systemic granulomatous disease of unknown etiology [1]. The primary impacted organ are the lungs in > 90% of the patients, but virtually any organ can be affected [2]. In Sweden, a prevalence of 160 cases per 100,000 individuals has been estimated [3]. In 20–40% of the cases the disease may start suddenly with an acute manifestation known as Löfgren’s syndrome (LS), with high fever, erythema nodosum and/or bilateral ankle arthritis, and bilateral hilar lymphadenopathy [2, 4–6]. However, the majority will have an insidious onset with fatigue, dry cough, dyspnea, low-grade fever, chest pain and sometimes weight loss (non-LS). The pathogenesis is still unknown, but some evidence indicates that causative antigen(s) enter the host and are phagocytosed by antigen-presenting cells (APCs), predominantly macrophages or dendritic cells. The APCs subsequently present the antigen via human leukocyte antigen (HLA) class II molecules primarily to a restricted set of CD4^+^ T lymphocytes [7]. The development of sarcoidosis seems to involve the intricate balance in the trimolecular complex consisting of an antigen, HLA class II molecules and T-cell receptors [8]. A genetic influence is evident as increased familial occurrence as well different disease modes in different ethnic groups have been shown [9]. The strongest genetic association is found within the HLA alleles that clearly associate with disease risk and phenotype [10]. For example, patients with the HLA-DRB1*0301 allele are known to associate with LS and a good prognosis with complete resolution within 2 years without treatment [10–12].

In the absence of a causative agent, sarcoidosis remains a diagnosis of exclusion of other diseases. There are no definitive diagnostic blood or radiologic imaging tests specific for the disorder, but there are several tests that aid in diagnosis [4], such as increased levels of serum ACE [13], chitotriosidase [14] or sIL-2R [15]. A cell pattern typical of lymphocytic alveolitis and a T-cell CD4^+^/CD8^+^ ratio > 3.5 have also been recognized [16]. However, no single biomarker of sarcoidosis has been reported that has sufficient specificity, sensitivity and reproducibility to reliably diagnose the disease [16]. Given the need for a molecular signature of the disease, this study was designed to investigate potential dysregulations of lipid mediators in patients with sarcoidosis. Lipid mediators are biologically active molecules that possess multiple physiological roles including cell structure, innate immune defense, homeostasis and in particular play a distinct role in inflammatory processes [17]. The role of lipid mediators in the etiology and pathology of respiratory diseases has been demonstrated, including asthma [18, 19], COPD [20] and cystic fibrosis [21]. Accordingly, in this study, three lipid mediator classes, eicosanoids, endocannabinoids and sphingolipids, were quantified in bronchoalveolar lavage (BAL) fluid from sarcoidosis patients and healthy controls. To our knowledge, the current study is the first to perform metabolic profiling of lipid mediators in newly diagnosed sarcoidosis.

## Methods

### Study population, bronchoscopy and bronchoalveolar lavage

This cross-sectional study consisted of 41 patients with sarcoidosis and 16 healthy volunteers as controls. Due to its influence upon lipid mediator levels [22], smoking was set as an exclusion criterion. All patients were referred to the Respiratory Medicine Unit at the Karolinska University Hospital (Stockholm, Sweden) on the suspicion of sarcoidosis for diagnostic investigations, including bronchoscopy and the morning collection of bronchoalveolar lavage (BAL) fluid as previously described [23]. The investigations were performed as close to onset of symptoms as possible and as soon as sarcoidosis was suspected. All patients were HLA-typed (Additional file 1) and diagnosed with sarcoidosis through typical clinical and radiographic manifestations, findings at bronchoscopy with biopsies revealing granuloma (not required for LS) and/or BAL fluid including an elevated CD4/CD8-ratio (> 3.5). Diagnoses were in accordance with the criteria established by the World Association of Sarcoidosis and other Granulomatous Disorders (WASOG) [24]. Chest radiography was classified as stage I (bilateral hilar lymphadenopathy, BHL) and stage II (BHL with parenchymal infiltrates). Lung function was analyzed with regard to FEV_1_ and FVC. All patients had symptoms compatible with an active disease, two of them were on prednisolone treatment at the time of BAL sampling, while four patients were on treatment with local steroids. Only two patients declared use of non-steroid anti-inflammatory drugs (NSAID) at the time of sampling. Healthy controls were recruited by announcement on a website used for recruiting healthy volunteers at the Karolinska University Hospital. A clinical examination was performed and blood was drawn for analyses of CRP, blood status and electrolytes. All volunteers had a normal chest X-ray at least 3 weeks prior to bronchoscopy and none showed signs of infection. Written informed consent was obtained from all subjects. None of the subjects had signs of any airway infection or allergy symptoms at the time of BAL sampling. Subjects diagnosed with asthma, COPD, and other lung diseases, or other inflammatory conditions were excluded from the study. The Regional Ethical Review Board in Stockholm approved the study. Samples were aliquoted and stored at -80 °C until analysis. On the day of extraction, after thawing at 4 °C (n_max_ = 24), samples were aliquoted for independent extractions of eicosanoids and endocannabinoids (Section 2.3.1) and sphingolipids (Section 2.3.2).

### Chemical standards

Eicosanoids and endocannabinoids were purchased from Cayman Chemical (Ann Arbor, USA), with the exception of 12(13)-EpODE, 9,10,13-TriHOME and 9,12,13-TriHOME from Larodan (Solna, Sweden) and LTB_5_ from Enzo (Solna, Sweden). Sphingolipids were purchased from Avanti Polar Lipids (Alabaster, USA). A complete list of lipid mediator nomenclature is provided in Additional file 2. Methanol, acetonitrile, isopropanol, acetic and formic acid were of LC-MS grade from Fischer Scientific (Stockholm, Sweden). Ethyl acetate and chloroform were purchased from Sigma-Aldrich (St. Louis, USA).

### Sample extraction

For eicosanoids and endocannabinoids, a volume of 3 mL (*n* = 53) and a minimum volume of 2.5 mL (*n* = 4) of BAL fluid was spiked with 10 μL of isotopically labeled internal eicosanoid and endocannabinoid standards (Additional file 3) and extracted with HLB Oasis solid phase extraction columns (3 cc/60 mg, Waters) as previously described [22]. Detailed information on the extraction is provided in Additional file 4.

For sphingolipids, a volume of 200 μL of BAL fluid in Eppendorf tubes were spiked with 10 μL of sphingolipid internal standards (Additional file 3) and subjected to a modified Bligh & Dyer extraction as previously described [25]. Detailed information on the extraction is provided in the Additional file 4.

### LC-MS/MS analysis

Eicosanoids, endocannabinoids and sphingolipids were screened using 3 separate targeted liquid chromatography tandem mass spectrometry (LC-MS/MS) lipid mediator platforms. Chromatographic separation was performed on an Acquity™ UPLC system from Waters (Milford, MA, USA). Detection was carried out on a Xevo TQ-S (Waters) mass spectrometer with electrospray ionization operating in selected reaction monitoring (MRM) mode. Detailed information of the separation and general MS conditions is provided in Additional file 5. Retention times and SRM transitions for each compound can be found elsewhere [22, 25, 26].

### Statistical analysis

Values below the lowest limit of quantification (LLOQ) were replaced by the 25% of the lowest detected concentration for that compound (Additional file 1). Univariate statistical analyses were performed using R (version 3.4.0). Fold changes were calculated by using the median between groups. A two-tailed Mann-Whitney U-test was used for comparisons between two groups. Multiple hypothesis testing was controlled according to Storey (q-value) using the QVALUE software [27]. A q-value lower than 5% was considered statistically significant. Correlation analyses were performed using Spearman’s correlation (two-tailed). Trends in the shifts of lipid mediators with chest radiography stages were evaluated using the Cochran-Armitage trend test for linearity. The median value among the healthy controls for each lipid mediator was used as a cut-off limit for all three groups and all trend-based *p*-values were one-tailed.

## Results

### Demographics

Cohort demographics are summarized in Table 1. A total of 21 subjects had LS and the patients presented with lower FEV_1_ and FVC scores relative to controls. No differences were observed in lung function between LS and non-LS patients or with regard to radiographic stage.

**Table 1:** Patient demographics from healthy controls and patients with sarcoidosis included in the current study. Demographics of patients with sarcoidosis according to the presence or absence of Löfgren’s syndrome and X-ray stage are also presented

	Healthy control	Sarcoidosis (All patients)	Sarcoidosis non-Löfgren	Sarcoidosis Löfgren	Sarcoidosis X-ray stage I	Sarcoidosis X-ray stage II
Number	16	41	20	21	22	19
Age, years	23 [23–25]	40 [33–47]^e^	43 [33–52]	40 [35–43]	41 [36–48]	40 [32–46]
Sex, female/male	8/8	15/26	7/13	8/13	7/15	8/11
CD4/CD8	1.8 [1.4–2.7]^a^	8.7 [4.8–11.5]^e^	7.0 [4.3–11.0]	9.6 [4.9–13.0]	9.7 [5.0–12.6]	7.3 [3.8–11.3]
X-ray stage, I/II	–	22/19	8/12	14/7	22/0	0/19
%FEV_1_	111 [101–118]	89 [81–100]^e^	89 [80–101]	89 [82–100]	88 [81–100]	89 [82–101]
%FVC	114 [109–117]	90 [81–99]^b,e^	91 [79–101]^c^	90 [82–96]^d^	90 [81–97]^e^	91 [81–100]^e^

Age, CD4/CD8, %FEV_1_ and %FVC are presented as median [Interquartile range]

^a^*n* = 13

^b^*n* = 28

^c^*n* = 16

^d^*n* = 12

^e^*p* < 0.05, 2 tailed t-test

### Lipid mediators in BAL fluid

A total of 47 lipids (eicosanoids: *n* = 26, sphingolipids: *n* = 17 and endocannabinoids: *n* = 4) were consistently detected in the samples and are summarized in Additional file 6.

### Differences between controls and patients with sarcoidosis

**Fig. 1:**
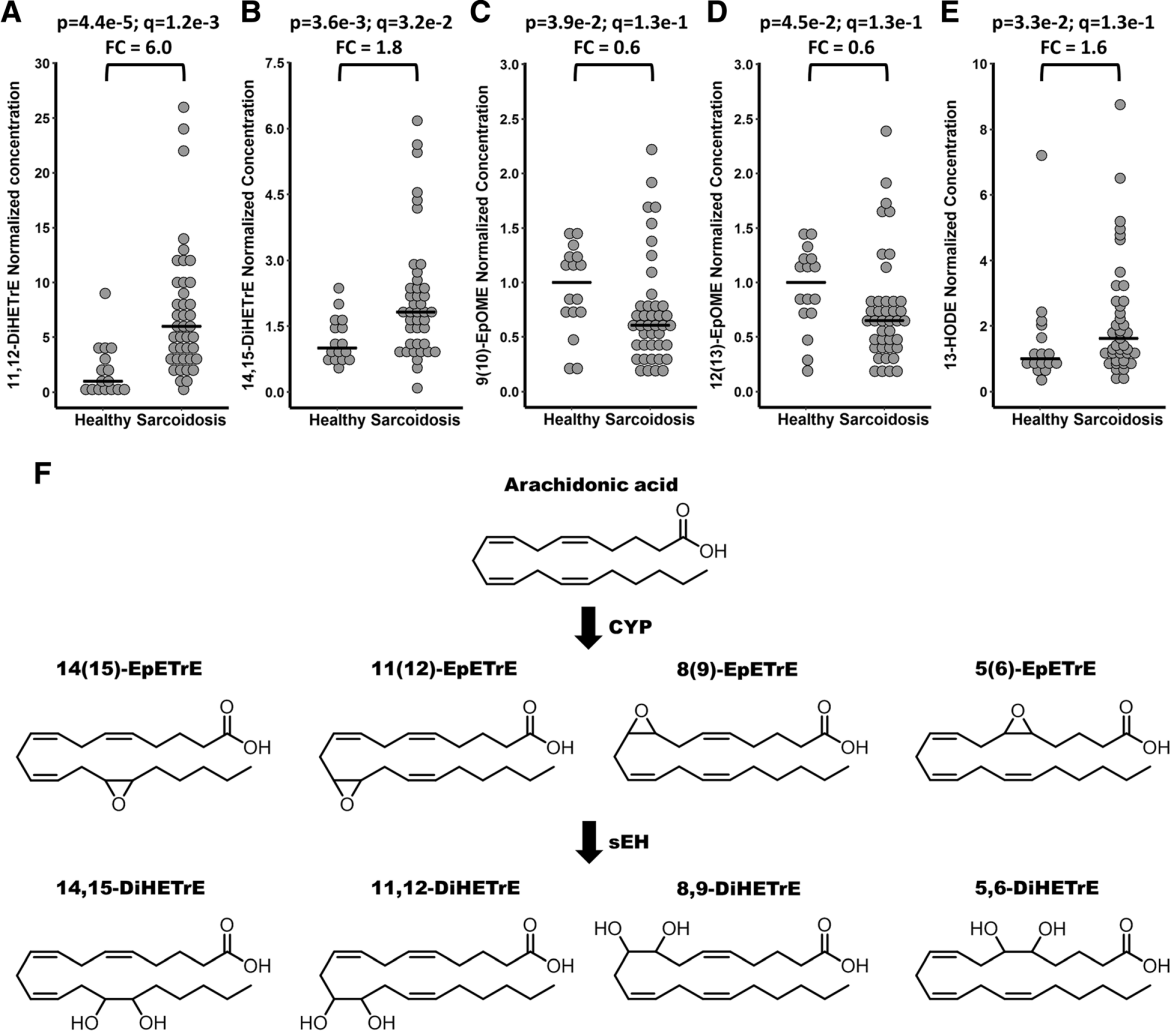
Eicosanoids displaying differential concentrations between healthy controls (*n* = 16) and patients with sarcoidosis (*n* = 41) (**a**-**e**) and biosynthetic pathway for production of DiHETrE from arachidonic acid (**f**). For graphical visualization, each value was normalized to the median concentration of the compound in the healthy group (Median of the healthy group = 1). Each dot represents an individual. The horizontal line indicates the median concentration in the group. *p* = Mann-Whitney U-test (two-tailed); q = Storey q-value; FC = Median fold change (Median sarcoidosis / Median healthy)

A total of 2 eicosanoids differed (q < 0.05) between controls and patients with sarcoidosis (Fig. 1 a, b, Additional file 6). The arachidonic acid-derived (Fig. 1f) 11,12-DiHETrE (*p* = 4.4 × 10^− 5^, q = 1.2 × 10^− 3^) and its regioisomer 14,15-DiHETrE (*p* = 3.6 × 10–3, q = 3.1 × 10–2) were increased (6.0- and 1.8-fold median change, respectively) in patients with sarcoidosis relative to controls. Additionally, three linoleic acid-derived eicosanoids, 13-HODE (*p* = 3.3 × 10^− 2^, q = 1.2 × 10^− 1^, FC = 1.6), 9(10)-EpOME (*p* = 3.9 × 10^− 2^, q = 1.2 × 10^− 1^, FC = 0.6) and 12(13)-EpOME (*p* = 4.5 × 10^− 2^, q = 1.2 × 10^− 1^, FC = 0.7) were dysregulated in patients with sarcoidosis relative to controls (*p* < 0.05), but were above the FDR threshold (Fig. 1 c-e).

**Fig. 2:**
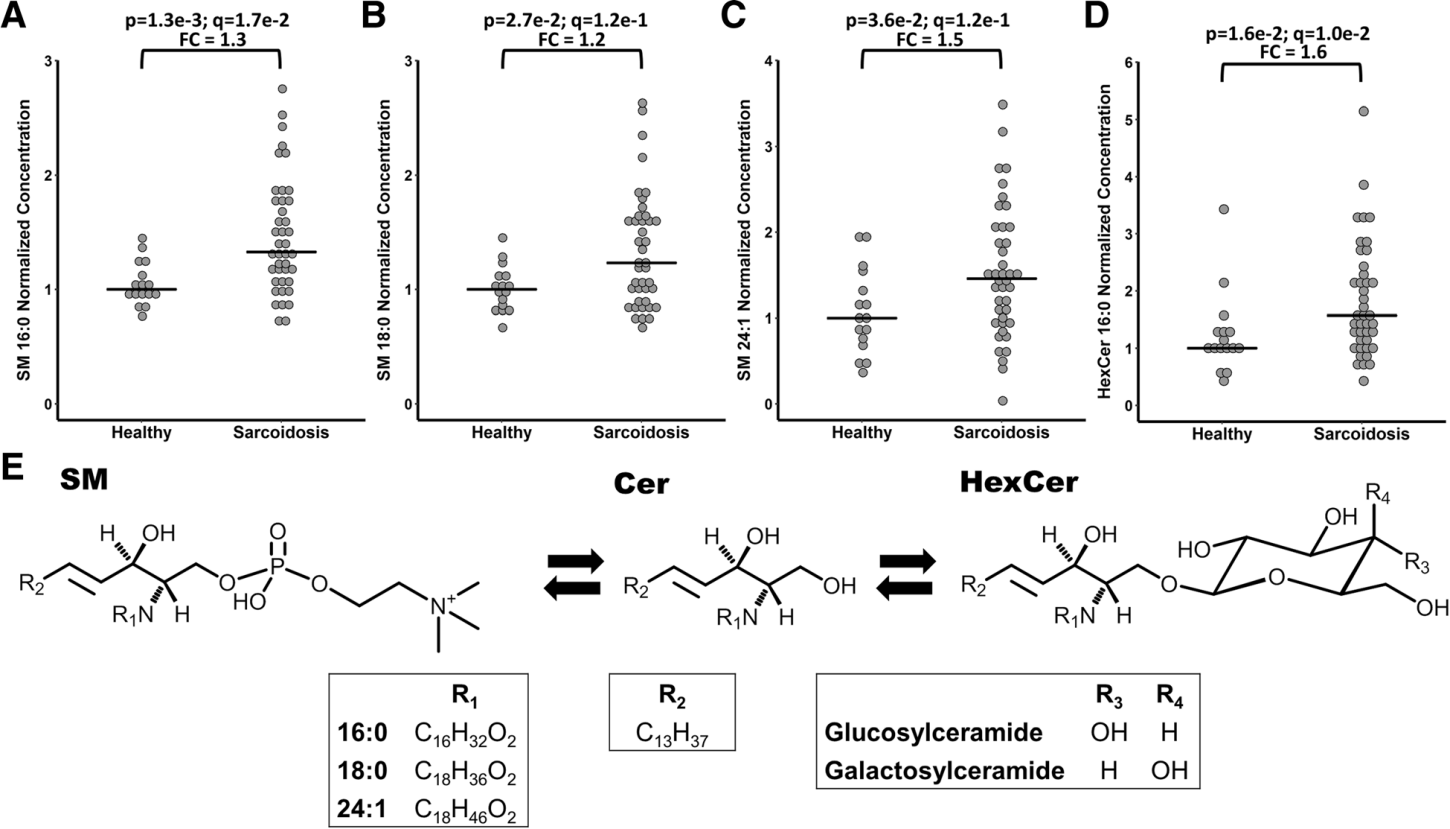
Sphingolipids displaying differential concentrations between healthy controls and patients with sarcoidosis. (**a**-**d**) Representation of the sphingolipid pathway (**e**). For graphical visualization, each value was normalized to the median concentration of the compound in the healthy group. Each dot represents an individual. The horizontal line indicates the median concentration in the group (Median of the healthy group = 1). *p* = Mann-Whitney *p*-value (two-tailed); q = Storey q-value; FC = Median fold change (Median sarcoidosis / Median healthy); SM = Sphingomyelin; Cer = Ceramide; HexCer = Hexosylceramide

One sphingolipid (SM 16:0 [*p* = 1.3 × 10^− 3^, q = 1.7 × 10^− 2^, FC = 1.3] was found increased in patients with sarcoidosis relative to controls. Additionally two sphingomyelins (SM 18:0; *p* = 2.7 × 10^− 2^, q = 1.3 × 10^− 1^, FC = 1.2 and SM 24:1; p = 3.6 × 10^− 2^, q = 1.3 × 10^− 1^, FC = 1.5), and one hexosylceramide (HexCer 16:0; *p* = 1.6 × 10^− 2^, q = 1.0 × 10^− 1^, FC = 1.6) (Fig. 2), were elevated in patients with sarcoidosis relative to controls (*p* < 0.05), but were above the FDR threshold.

There were no differences (p < 0.05) between patients with sarcoidosis and healthy controls in endocannabinoid levels (Additional file 6). None of the compounds that were dysregulated between the groups correlated with age in either the healthy or sarcoidosis groups (Additional file 7).

### Lipid mediator associations with signs of disease staging and activity

**Fig. 3:**
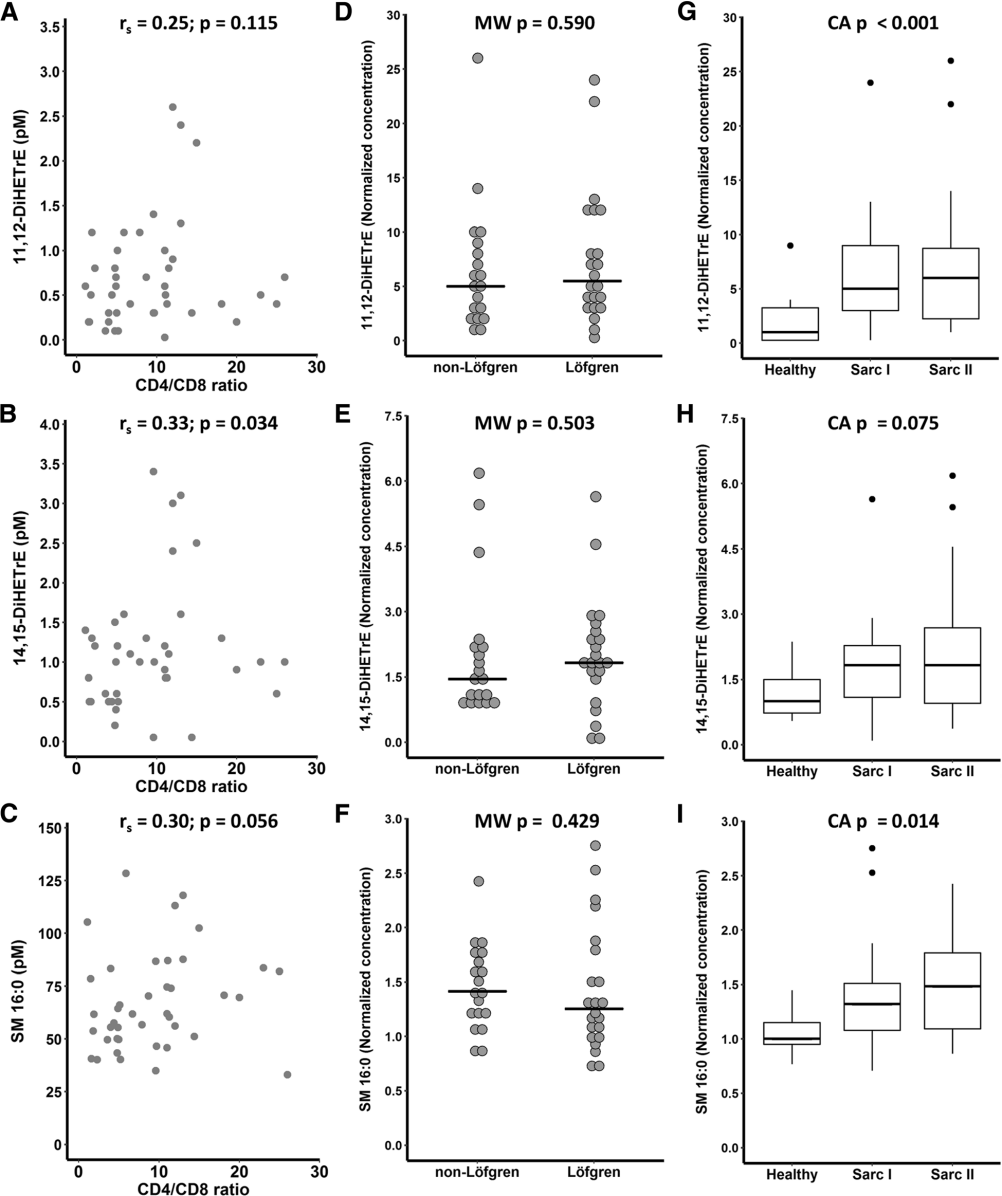
Associations of lipids differentially expressed in patients with sarcoidosis (q < 0.05) and clinical measurements. (**a**-**c**) Spearman’s correlation with CD4/CD8 ratio. (**d**-**f**) Relative levels in sarcoidosis patients with and without Löfgren’s syndrome syndrome. (**g**-**i**) Levels according to radiological stage. For graphical visualization, each value was normalized to the median concentration of the compound in the healthy group. CA = Cochran-Armitage test; DiHETrE = Dihydroxyeicosatrienoic acid; HC = healthy control; MW = Mann-Whitney (two-tailed); r_s_ = Spearman’s rho; Sarc I = sarcoidosis radiologic stage 1; Sarc II = sarcoidosis radiologic stage 2; SM = Sphingomyelin. See Figs. 1 and 2 for compound structures

**Fig. 4:**
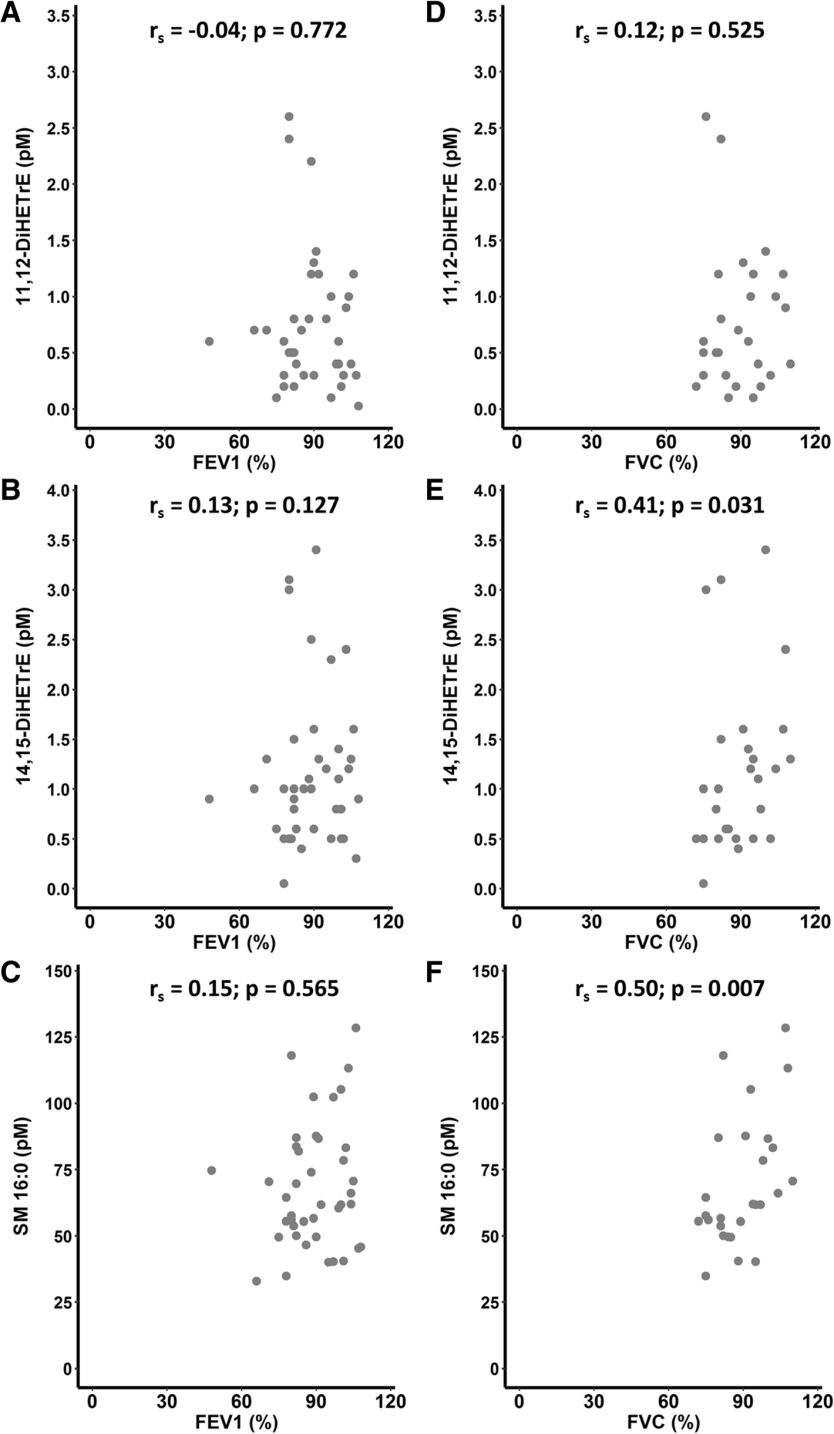
Associations of lipids differentially expressed in patients with sarcoidosis (q < 0.05) and pulmonary function. (**a**-**c**) Spearman’s correlation with (**a**-**c**) FEV1 (%) and (**d**-**f**) FVC (%). r_s_ = Spearman’s rho. DiHETrE = Dihydroxyeicosatrienoic acid; SM = Sphingomyelin. See Figs. 1 and 2 for compound structures

For lipid mediators differing between controls and patients with sarcoidosis after false discovery rate adjustment (q < 0.05), associations with LS, CD4/CD8 ratio, chest radiograph stage, %FEV_1_ and %FVC were performed (Figs. 3 and 4). Due to the exploratory nature of this study, associations of lipid mediators with *p* < 0.05 were also performed and are presented in Additional file 8. For these compounds, a weak correlation was found between levels of 14,15-DiHETrE (r_s_ = 0.33, *p* = 3.4x10E^− 2^) and CD4/CD8 ratio (Fig. 3b). No differences were found between patients with or without Löfgren syndrome (Fig. 3d, e). Levels of 11,12-DiHETrE (*p* = 2.2 × 10^− 4^) and SM 16:0 (*p* = 2.5 × 10^− 2^) increased with radiography stage according to the Cochran-Armitage test (Fig. 3g and i). Weak associations with %FVC were found between levels of 14,15-DiHETrE (r_s_ = 0.41, *p* = 3.1x10E^− 2^) and SM 16:0 (r_s_ = 0.50, *p* = 7.0x10E^− 3^) (Fig. 4).

## Discussion

Given the known role of lipid mediators in inflammation [18, 19], and specifically pro-inflammatory eicosanoids and sphingolipids [17], in respiratory disease, this exploratory study was designed to profile these lipid mediators in BAL fluid of patients with sarcoidosis. We hypothesized that the findings would identify molecular descriptors of the disease. In addition, it was expected that increased knowledge of the dynamics surrounding lipid mediator production in sarcoidosis would provide improved understanding of disease processes in the organ most commonly affected by the disease.

Multiple lipid mediators were quantified in the BAL fluid, further supporting that this material is useful for monitoring biochemical process in the lung. The primary observed shifts in lipid mediators were increases in the BAL fluid concentrations of 11,12-DiHETrE and 14,15-DiHETrE with sarcoidosis (Fig. 1). Interestingly, the concentration of 11,12-DiHETrE also increased with radiography stages I and II in patients with sarcoidosis. These compounds are formed via the cytochrome P450-mediated oxidation of arachidonic acid to the corresponding epoxyeicosatrienoic acids (EpETrEs): 11(12)-EpETrE and 14(15)-EpETrE [28]. Out of the 4 regioisomers of epoxyeicosatrienoic acids screened (Fig. 1f), only 11(12)-EpETrE was observed, but this compound was not altered in patients. The EpETrEs can be metabolized via multiple pathways [29], with the soluble epoxide hydrolase (sEH) being the dominant pathway in most tissues [28]. The EpETrEs are generally considered to exert anti-inflammatory properties, while the DiHETrEs are pro-inflammatory [30, 31]. These opposing effects have been observed in models of pulmonary diseases [32]. A combination of sEH inhibitors and EpETrE treatment reduced tobacco smoke-induced inflammation, with an concomitant decrease in DiHETrE in a smoking rat model, demonstrating that sEH inhibition can attenuate at least part of the acute inflammation associated with tobacco exposure [32]. Accordingly, the observed increase in DiHETrEs suggests an inflammatory response, which is also in agreement with other reported markers of an ongoing inflammation in sarcoidosis [16]. The sEH pathway has also been suggested as a potential therapeutic target in cystic fibrosis [21], COPD [22] and asthma [33]. Clinical trials are currently underway to investigate how cytochrome P450 epoxygenase pathway enzymes, including the sEH, affect macrophage function in the lungs and inflammatory responses (clinicaltrials.gov NCT02743468) as well as in COPD (clinicaltrials.gov NCT01762774). Interestingly, the linoleic acid-derived 9(10)- and 12(13)-EpOME (structural analogs of the arachidonic acid-derived EpETrEs) were decreased in patients with sarcoidosis. Though these compounds are also metabolized via the sEH to form the corresponding vicinal diols, no differences were found between patients and controls in the BAL fluid levels of the downstream products (9,10-DiHOME and 12,13-DiHOME, respectively). These data suggest that the lipid mediator dysregulations in sarcoidosis are specific for the arachidonic acid pathway, as opposed to COPD [22], which demonstrates dysregulation in the linoleic acid-derived mediators.

Changes in sphingolipid metabolism involved multiple lipid species, including SM (16:0, 18:0, 24:1) and HexCer (16:0). Sphingomyelins have been previously reported to increase in the BAL fluid of sarcoidosis patients [34]. These sphingolipids are biochemically linked, with ceramides acting as the intermediate for the formation of both sphingomyelins and hexosylceramides (Fig. 2e). Sphingolipids have an essential role as structural components of membranes, but also serve as critical signaling molecules [35]. They are also involved in the pathophysiology of many diseases including microbial infections [36], Alzheimer’s disease [37] and respiratory disease [38]. For example, in asthma the ORMDL genes have been identified as a risk factor for childhood asthma [39]. Moreover several biosynthetic intermediates in the sphingolipid biosynthesis, including ceramides and sphingosine-1-phosphate have been shown to affect immune-cell trafficking, mast cell degranulation and airway hyper-responsiveness in asthma-associated inflammatory processes [40–42]. In the current study, a weak association between SM 16:0 and radiography stage was observed. Further studies should include patients with advanced disease stages to more closely investigate possible associations of sphingomyelins with disease development.

There are some limitations in this study. First, there is an age imbalance between sarcoidosis patients and healthy controls. However, none of the dysregulated metabolites was associated with age in patients or controls. Two patients were on prednisolone treatment and four more were on local steroids at the time of sampling, but they did not exhibit different profiles in the reported lipid mediators (data not shown), which does not suggest a strong effect of steroid treatment upon lipid mediator profiles. Additionally, patients with more advanced stages of the disease would be needed in order to confirm the association of the identified markers with the sarcoidosis radiological stage. On the other hand, this study explored the lipid mediator profiles close to disease onset, minimizing any potential effects associated with ongoing treatment, and highlighting disease-specific processes. In addition, the specificity of the observed dysregulations in lipid mediator levels need to be established relative to other respiratory diseases. Lastly, an obstacle to working with BAL fluid is the lack of clear protocols to perform data normalization. Previous attempts have been made to normalize the data using for example recovery volume [43] or metabolite markers [44], but there are of as yet no commonly accepted protocols.

## Conclusions

This is the first study to profile lipid mediators in sarcoidosis, providing useful information on the levels of these potent biological mediators in this disease. The findings suggest that arachidonic acid-derived products of the sEH pathway as well as some sphingolipid species (particularly SM 16:0) may serve as molecular descriptors of sarcoidosis. Of particular interest are the observations that the primary altered pathways are similar to those observed in other respiratory diseases, including asthma, COPD, and cystic fibrosis, suggesting that the diseases share a similar inflammatory component. The predominant shifts were in sEH products of arachidonic acid metabolism, further stressing the potential role of this pathway in the inflammatory process in lung pathology and highlighting the targeting of this enzyme for disease treatment. It is of particular interest that these sEH products also correlated with radiological stage, further strengthening their association with more advanced disease. These findings suggest that the sEH pathway should be further investigated in sarcoidosis, both from the standpoint of potentially being involved in disease etiology and representing a novel therapeutic target for disease control.

## Additional files

Additional file 1: **Data S1.** Summary of data used in the analysis. (XLSX 31 kb)

Additional file 2: **Table S2.** Nomenclature for the compounds screened in the samples. Compounds reproducibly detected in samples and submitted to data analysis are highlighted in bold. (DOCX 196 kb)

Additional file 3: **Table S3.** Internal standards for each of the three LC-MS/MS lipid mediator platforms. (PDF 78 kb)

Additional file 4: **Appendix S1.** Detailed procedures employed for the extraction of eicosanoids, endocannabinoids and sphingolipids. (PDF 11 kb)

Additional file 5: **Appendix S2.** Chromatographic and general MS conditions employed in the detection of eicosanoids, sphingolipids and endocannabinoids. (PDF 97 kb)

Additional file 6: **Table S4.** Results for the comparisons of lipid mediator levels between healthy (*n* = 16) and sarcoidosis (*n* = 41) groups. Values have been normalized to the median value of the healthy group. Compounds with *p* < 0.05 are highlighted in bold. (PDF 71 kb)

Additional file 7: **Table S5.** Spearman’s correlations between lipid mediators and age for healthy controls and patients with sarcoidosis. Compounds with *p* < 0.05 are highlighted in bold. (PDF 39 kb)

Additional file 8: **Table S6.** Results of the Cochran-Armitage test with radiography stage in sphingolipids significantly differing (*p* < 0.05) between patients and controls. Direction of the null hypothesis is indicated for each compound. Compounds with *p* < 0.05 are highlighted in bold. (PDF 79 kb)

## Abbreviations

APCs: Antigen presenting cells
BAL: Bronchoalveolar lavage
BHL: Bilateral hilar lymphadenopathy
DiHETrE: Dihydroxyeicosatrienoic acid
EpOME: Epoxyoctadecenoic acid
FC: Fold change
GalCer: Galactosylceramide
GluCer: Flucosylceramide
HexCer: Hexosylceramide
HLA: Human leukocyte antigen
HODE: Hydroxyoctadecadienoic acid
LC-MS/MS: Liquid chromatography tandem mass spectrometry
LLOQ: Lowest limit of quantification
sEH: Soluble epoxyde hydrolase
SM: Sphingomyelin
SRM: Selected reaction monitoring
