# Supplementary material for: Soluble epoxide hydrolase derived lipid mediators are elevated in bronchoalveolar lavage fluid from patients with sarcoidosis: a cross-sectional study
Source: Respir Res. 2018 Dec 3;19:236. doi: 10.1186/s12931-018-0939-0 (PMC6276236; doi:10.1186/s12931-018-0939-0)
Supplement: Supplementary file 2 — Table S2. Nomenclature for the compounds screened in the samples. Compounds reproducibly detected in samples and submitted to data analysis are highlighted in bold. (DOCX 196 kb) [file 12931_2018_939_MOESM2_ESM.pdf]

Table S1. Nomenclature for the compounds screened in the samples. Compounds reproducibly detected in samples and submitted to data analysis are highlighted in bold.

Abbreviation	Common name	Systematic name
Eicosanoids		
**PGD$_2$**	**prostaglandin D$_2$**	**9α.15S-dihydroxy-11-oxo-prosta-5Z.13E-dien-1-oic acid**
11-β-PGF$_{2\alpha}$	9α.11β-prostaglandin F$_{2\alpha}$	9α.11β.15S-trihydroxy-prosta-5Z.13E-dien-1-oic acid
PGJ$_2$	prostaglandin J$_2$	11-oxo-15S-hydroxy-prosta-5Z.9.13E-trien-1-oic acid
15-deoxy-$\Delta^{12.14}$- PGJ$_2$	15-deoxy-$\Delta^{12.14}$-prostaglandin J$_2$	11-oxo-prosta-5Z.9.12E.14E-tetraen-1-oic acid
PGE2	prostaglandin E$_2$; dinoprostone	9-oxo-11α.15S-dihydroxy-prosta-5Z.13E-dien-1-oic acid
PGF$_{2\alpha}$	prostaglandin F$_{2\alpha}$; dinoprost	9α.11α.15S-trihydroxy-prosta-5Z.13E-dien-1-oic acid
6-keto-PGF$_{1\alpha}$	6-keto-prostaglandin F$_{1\alpha}$	6-oxo-9α.11α.15S-trihydroxy-prost-13E-en-1-oic acid
TxB$_2$	thromboxane B$_2$	9α.11.15S-trihydroxythromba-5Z.13E-dien-1-oic acid
12-HHTrE	12(S)-HHT	12S-hydroxy-5Z.8E.10E-heptadecatrienoic acid
PGB$_2$	prostaglandin B$_2$	9-oxo-15S-hydroxy-prosta-5Z.8(12).13E-trien-1-oic acid
8-isoPGE$_2$	8-iso-prostaglandin E$_2$	9-oxo-11α.15S-dihydroxy-(8β)-prosta-5Z.13E-dien-1-oic acid
**LTB$_4$**	**leukotriene B$_4$**	**5S.12R-dihydroxy-6Z.8E.10E.14Z-eicosatetraenoic acid**
6-trans-LTB$_4$	6-trans-leukotriene B4	5S.12R-dihydroxy-6E.8E.10E.14Z-eicosatetraenoic acid
20-hydroxy-LTB$_4$	20-hydroxy-leukotriene B4	5S.12R.20-trihydroxy-6Z.8E.10E.14Z-eicosatetraenoic acid
20-carboxy-LTB$_4$	20-carboxy-leukotriene B4	5S.12R-dihydroxy-6Z.8E.10E.14Z-eicosatetraene-1.20-dioic acid
**5-HETE**	**(±)5-HETE**	**(±)5-hydroxy-6E.8Z.11Z.14Z-eicosatetraenoic acid**
8-HETE	(±)8-HETE	(±)8-hydroxy-5Z.9E.11Z.14Z-eicosatetraenoic acid
**9-HETE**	**(±)9-HETE**	**(±)-9-hydroxy-5Z.7E.11Z.14Z-eicosatetraenoic acid**
**11-HETE**	**(±)11-HETE**	**(±)11-hydroxy-5Z.8Z.12E.14Z-eicosatetraenoic acid**
12-HETE	(±)12-HETE	(±)12-hydroxy-5Z.8Z.10E.14Z-eicosatetraenoic acid
**15-HETE**	**(±)15-HETE**	**(±)15-hydroxy-5Z.8Z.11Z.13E-eicosatetraenoic acid**
20-HETE	20-hydroxy arachidonic acid	20-hydroxy-5Z.8Z.11Z.14Z-eicosatetraenoic acid
5-KETE	5-oxoETE	5-oxo-6E.8Z.11Z.14Z-eicosatetraenoic acid
12-KETE	12-oxoETE	12-oxo-5Z.8Z.10E.14Z-eicosatetraenoic acid
**15-KETE**	**15-oxoETE**	**15-oxo-5Z.8Z.11Z.13E-eicosatetraenoic acid**
**5(6)-EpETrE**	**(±)5(6)-EET**	**(±)5(6)-epoxy-8Z.11Z.14Z-eicosatrienoic acid**
8(9)-EpETrE	(±)8(9)-EET	(±)8(9)-epoxy-5Z.11Z.14Z-eicosatrienoic acid
**11(12)-EpETrE**	**(±)11.12-EET**	**(±)11(12)-epoxy-5Z.8Z.14Z-eicosatrienoic acid**
14(15)-EpETrE	(±)14(15)-EET	(±)14(15)-epoxy-5Z.8Z.11Z-eicosatrienoic acid
5.6-DiHETrE	(±)5.(6)-DHET	(±)5.6-dihydroxy-8Z.11Z.14Z-eicosatrienoic acid
8.9-DiHETrE	(±)8.9-DHET	(±)8.9-dihydroxy-5Z.11Z.14Z-eicosatrienoic acid
**11.12-DiHETrE**	**(±)11.12-DHET**	**(±)11.12-dihydroxy-5Z.8Z.14Z-eicosatrienoic acid**
**14.15-DiHETrE**	**(±)14.15-DHET**	**(±)14.15-dihydroxy-5Z.8Z.11Z-eicosatrienoic acid**
5.6-DiHETE	5.6-DiHETE	5.6-dihydroxy-8Z.11Z.14Z.17Z-eicosatetraenoic acid
5.15-DiHETE	5(S).15(S)-DiHETE	5S.15S-dihydroxy-6E.8Z.10Z.13E-eicosatetraenoic acid
8.15-DiHETE	8(S).15(S)-DiHETE	8S.15S-dihydroxy-5Z.9E.11Z.13E-eicosatetraenoic acid
LXA$_4$	5(S).6(R)-lipoxin A4	5S.6R.15S-trihydroxy-7E.9E.11Z.13E-eicosatetraenoic acid
LXB$_4$	5(S).14(R)-lipoxin B4	5S.14R.15S-trihydroxy-6E.8Z.10E.12E-eicosatetraenoic acid
**9-HODE**	**(±)9-HODE**	**(±)-9-hydroxy-10E.12Z-octadecadienoic acid**
**13-HODE**	**(±)13-HODE**	**(±)-13-hydroxy-9Z.11E-octadecadienoic acid**
9-KODE	9-oxoODE	9-oxo-10E.12Z-octadecadienoic acid
**13-KODE**	**13-oxoODE**	**13-oxo-9Z.11E-octadecadienoic acid**
9.10.13-TriHOME	9.10.13-TriHOME	9(S).10(S).13(S)-trihydroxy-11(E)-octadecenoic Acid
9.12.13-TriHOME	9.12.13-TriHOME	9(S).12(S).13(S)-trihydroxy-10(E)-octadecenoic Acid
**EKODE**	**trans-EKODE-(E)-Ib**	**9-oxo-11-(3-pentyl-2-oxiranyl)-10E-undecenoic acid**
**9(10)-EpOME**	**leukotoxin**	**(±)9(10)-epoxy-12Z-octadecenoic acid**
**12(13)-EpOME**	**iso-leukotoxin**	**(±)12(13)epoxy-9Z-octadecenoic acid**
**9.10-DiHOME**	**leukotoxin diol**	**(±)9(10)-dihydroxy-12Z-octadecenoic acid**
**12.13-DiHOME**	**isoleukotoxin diol**	**12.13-dihydroxy-9Z-octadecenoic acid**
**12(13)-EpODE**	**(±)12(13)-EpODE**	**(±)-cis-12.13-Epoxy-9(Z).15(Z)-octadecadienoic Acid**
PGD$_1$	prostaglandin D$_1$	9α.15S-dihydroxy-11-oxo-prost-13E-en-1-oic acid
PGE$_1$	prostaglandin E$_1$	9-oxo-11α.15S-dihydroxy-prost-13E-en-1-oic acid
8-HETrE	8(S)-HETrE	5S-hydroxy-9E.11Z.14Z-eicosatrienoic acid
8-HETrE	8(S)-HETrE	8S-hydroxy-9E.11Z.14Z-eicosatrienoic acid

**15-HETrE**	**15(S)-HETrE**	**15S-hydroxy-8Z.11Z.13E-eicosatrienoic acid**
9-HOTrE	9(S)-HOTrE	9S-hydroxy-10E.12Z.15Z-octadecatrienoic acid
**13-HOTrE**	**13(S)-HOTrE**	**13S-hydroxy-9Z.11E.15Z-octadecatrienoic acid**
9-KOTrE	9-KOTE	9-oxo-10E.12Z.15Z-octadecatrienoic acid
PGD_3	prostaglandin D_3	9α.15S-dihydroxy-11-oxo-prosta-5Z.13E.17Z-trien-1-oic acid
PGE_3	prostaglandin E_3	9-oxo-11α.15S-dihydroxy-prosta-5Z.13E.17Z-trien-1-oic acid
LTB_5	leukotriene B_5	5S.12S-dihydroxy-6Z.8E.14Z.17Z-eicosapentanenoic acid
**5-HEPE**	**(±)5-HEPE**	**(±)-5-hydroxy-6E.8Z.11Z.14Z.17Z-eicosapentaenoic acid**
8-HEPE	(±)8-HEPE	(±)-8-hydroxy-5Z.9E.11Z.14Z.17Z-eicosapentaenoic acid
9-HEPE	(±)9-HEPE	(±)-9-hydroxy-5Z.7E.11Z.14Z.17Z-eicosapentaenoic acid
11-HEPE	(±)11-HEPE	(±)-11-hydroxy-5Z.8Z.12E.14Z.17Z-eicosapentaenoic acid
12-HEPE	(±)12-HEPE	(±)-12-hydroxy-5Z.8Z.10E.14Z.17Z-eicosapentaenoic acid
15-HEPE	(±)15-HEPE	(±)-15-hydroxy-5Z.8Z.11Z.13E.17Z-eicosapentaenoic acid
18-HEPE	(±)18-HEPE	(±)-18-hydroxy-5Z.8Z.11Z.14Z.16E-eicosapentaenoic acid
14(15)-EpETE	14.15-epoxy eicosatetraenoic Acid	(±)14(15)-epoxy-5Z.8Z.11Z.17Z-eicosatetraenoic acid
17(18)-EpETE	17.18-epoxy eicosatetraenoic Acid	(±)17(18)-epoxy-5Z.8Z.11Z.14Z-eicosatetraenoic acid
14.15-DiHETE	(±)14.15-DiHETE	(±)14.15-dihydroxy-eicosa-5.8.11.17-tetraenoic acid
17.18-DiHETE	(±)17.18-DiHETE	17.18-dihydroxy-5Z.8Z.11Z.14Z-eicosatetraenoic acid
LXA_5	lipoxin A_5	5S.6R.15S-trihydroxy-7E.9E.11Z.13E.17Z-eicosapentaenoic acid
8-HDoHE	(±)8-HDoHE	(±)8-hydroxy-4Z.6E.10Z.13Z.16Z.19Z-docosahexaenoic acid
11-HDoHE	(±)11-HDoHE	(±)11-hydroxy-4Z.7Z.9E.13Z.16Z.19Z-docosahexaenoic acid
**14-HDoHE**	**(±)14-HDoHE**	**(±)14-hydroxy-4Z.7Z.10Z.12E.16Z.19Z-docosahexaenoic acid**
**17-HDoHE**	**(±)17-HDoHE**	**(±)17-hydroxy-4Z.7Z.10Z.13Z.15E.19Z-docosahexaenoic acid**
16(17)-EpDPE	(±)16(17)-EpDPE	(±)16(17)-epoxy-4Z.7Z.10Z.13Z.19Z-docosapentaenoic acid
19(20)-EpDPE	(±)19(20)-EpDPE	(±)19(20)-epoxy-4Z.7Z.10Z.13Z.16Z-docosapentaenoic acid
10.17-DiHDoHE	protectin D_1	10(S).17(S)-dihydroxy-4Z.7Z.11E.13Z.15E.19Z-docosahexaenoic acid
19.20-DiHDPA	(±)19.20-DiHDPA	(±)19.20-dihydroxy-4Z.7Z.10Z.13Z.16Z-docosapentaenoic acid
RvD_1	resolvin D_1	7S.8R.17S-trihydroxy-4Z.9E.11E.13Z.15E.19Z-docosahexaenoic acid
17(R)-RvD_1	17(R)-resolvin D1	7S.8R.17R-trihydroxy-4Z.9E.11E.13Z.15E19Z-docosahexaenoic acid
RvD_2	resolvin D_2	7S.16R.17S-trihydroxy-4Z.8E.10Z.12E.14E.19Z-docosahexaenoic acid
RvE_1	resolvin E_1	5S.12R.18R-trihydroxy-6Z.8E.10E.14Z.16E-eicosapentaenoic acid
LTB_3	leukotriene B_3	5S.12R-dihydroxy-6Z.8E.10E-eicosatrienoic acid
**5-HETrE**	**5(S)-HETrE**	**5S-hydroxy-6E.8Z.11Z-eicosatrienoic acid**

Sphingolipids

**SM 12:0**	**Sphingomyelin C12:0**	**(2-{[(2S.3R)-3-hydroxy-2-[(1-hydroxydodecyl)amino]octadecyl phosphonato]oxy}ethyl)trimethylazanium**
**SM 16:0**	**Sphingomyelin C16:0**	**{[(2S.3R.4E)-2-hexadecanamido-3-hydroxyoctadec-4-en-1-yl]oxy}[2-(trimethylazaniumyl)ethoxy]phosphinic acid**
**SM 18:1**	**Sphingomyelin C18:1**	**[(E.2S.3R)-3-hydroxy-2-[[(Z)-octadec-9-enoyl]amino]octadec-4-enyl] 2-(trimethylazaniumyl)ethyl phosphate**
**SM 18:0**	**Sphingomyelin C18:0**	**[(E.2S.3R)-3-hydroxy-2-(octadecanoylamino)octadec-4-enyl] 2-(trimethylazaniumyl)ethyl phosphate**
**SM 24:1**	**Sphingomyelin C24:1**	**N-(15Z-tetracosenoyl)-sphing-4-enine-1-phosphocholine**
**SM 24:0**	**Sphingomyelin C24:0**	**N-(tetracosanoyl)-sphing-4-enine-1-phosphocholine**
Cer 12:0	Ceramide C12:0	N-(dodecanoyl)-sphing-4-enine
Cer 14:0	Ceramide C14:0	N-(tetradecanoyl)-sphing-4-enine
**Cer 16:0**	**Ceramide C16:0**	**N-(hexadecanoyl)-sphing-4-enine**
Cer 18:1	Ceramide C18:1	N-(9Z-octadecenoyl)-sphing-4-enine
**Cer 18:0**	**Ceramide C18:0**	**N-(octadecanoyl)-sphing-4-enine**
**Cer 20:0**	**Ceramide C20:0**	**N-(eicosanoyl)-sphing-4-enine**
**Cer 22:0**	**Ceramide C22:0**	**N-behenoyl-D-*erythro*-sphingosine**
**Cer 24:1**	**Ceramide C24:1**	**N-(15Z-tetracosenoyl)-sphing-4-enine**
**Cer 24:0**	**Ceramide C24:0**	**N-(tetracosanoyl)-sphing-4-enine**
DhCer 16:0	Dihydroceramide 16:0	N-palmitoyl-D-*erythro*-sphinganine
DhCer 24:0	Dihydroceramide C24:0	N-lignoceroyl-D-*erythro*-sphinganine
HexCer 12:0	Hexosylceramide C12:0	N-(dodecanoyl)-1-ß-hexosyl-sphing-4-enine
**HexCer 16:0**	**Hexosyllceramide C16:0**	**N-(hexadecanoyl)-1-ß- hexosyl-sphing-4-enine**
HexCer 18:1	Hexosylceramide C18:1	N-(9Z-octadecenoyl)-1-ß- hexosyl -sphing-4-enine
**HexCer 18:0**	**Hexosylceramide C18:0**	**N-(octadecanoyl)-1-ß- hexosyl -sphing-4-enine**
**HexCer 24:1**	**Hexosylceramide C24:1**	**N-(15Z-tetracosenoyl)-1-ß-hexosyl-sphing-4-ene**
LacCer 12:0	Lactosylceramide C12:0	N-(dodecanoyl)-1-b-lactosyl-sphing-4-enine

**LacCer 16:0**	**Lactosylceramide C16:0**	**N-Hexadecanoyl-lactosylceramide**
**LacCer 24:1**	**Lactosylceramide C24:1**	**N-(15Z-tetracosenoyl)-1-β-lactosyl-sphinganine**
LacCer 24:0	Lactosylceramide C24:0	N-(tetracosanoyl)-1-β-lactosyl-sphinganine
**Endocannabinoids**		
AEA	Arachidonoyl ethanolamine	5Z.8Z.11Z.14Z-eicosatetraenoylethanolamide
**PEA**	**Palmitoyl ethanolamide**	**N-(2-Hydroxyethyl)hexadecanamide**
EPEA	Eicosapentaenoyl ethanolamide	N-(2-hydroxyethyl)-5Z.8Z.11Z.14Z.17Z-eicosapentaenamide
D(h)EA	Docosahexaenoyl ethanolamide	N-(2-hydroxyethyl)-4Z.7Z.10Z.13Z.16Z.19Z-docosahexaenamide
**LEA**	**Linoleoyl ethanolamide**	**N-(2-hydroxyethyl)-9Z.12Z-octadecadienamide**
D(t)EA	Docosatetraenoyl ethanolamide	N-(2-hydroxyethyl)-7Z.10Z.13Z.16Z-docosatetraenamide
OEA	Oleoyl ethanolamide	(Z)-N-(2-Hydroxyethyl)octadec-9-enamide
SEA	Stearoyl ethanolamide	N-(2-hydroxyethyl)-octadecanamide
α-LINOLEA	α-linoleoyl ethanolamide	N-(2-hydroxyethyl)-9Z.12Z-octadecadienamide
ϒ-DIHOMOLEA	Dihomo- ϒ-linoleoyl ethanolamide	Cis.cis.cis-8.11.14-Eicosatrienoic acid
**AG**	**1+2-arachidonoyl ethanolamide**	**1.3-Dihydroxy-2-propanyl (5Z.8Z.11Z.14Z)-5.8.11.14-eicosatetraenoate**
**LG**	**1+2-linoleoyl ethanolamide**	**9Z.12Z-octadecadienoic acid. 2-glyceryl ester**
