# Supplementary material for: Soluble epoxide hydrolase derived lipid mediators are elevated in bronchoalveolar lavage fluid from patients with sarcoidosis: a cross-sectional study
Source: Respir Res. 2018 Dec 3;19:236. doi: 10.1186/s12931-018-0939-0 (PMC6276236; doi:10.1186/s12931-018-0939-0)
Supplement: Supplementary file 3 — Table S3. Internal standards for each of the three LC-MS/MS lipid mediator platforms. (PDF 78 kb) [file 12931_2018_939_MOESM3_ESM.pdf]

Table S2. Internal standards for each of the three LC-MS/MS lipid mediator platforms.

Internal standard	Concentration (nM)	Retention time (min)	Precursor ion (m/z)	Parent ion (m/z)	Product ion (m/z)
[$^{2}H_{4}$]-PGD_2	2.01×10^{2}	6.10	$[M-H]^{-}$	355.3	193
[2H4]-PGE2	2.01×10^{2}	5.85	[M-H]-	355.3	275
[$^{2}H_{4}$]-6-keto-$PGF_{1\alpha}$	2.00×10^{2}	4.35	$[M-H]^{-}$	373.2	167
[$^{2}H_{4}$]-PGB_2	2.01×10^{2}	8.00	$[M-H]^{-}$	337.2	179
[$^{2}H_{4}$]-TxB_2	2.00×10^{2}	5.22	$[M-H]^{-}$	373.2	173
[$^{2}H_{4}$]-LTB_4	2.00×10^{2}	9.60	$[M-H]^{-}$	339.3	153
[$^{2}H_{8}$]-5-HETE	2.01×10^{2}	14.15	$[M-H]^{-}$	327.2	116
[$^{2}H_{8}$]-15-HETE	2.01×10^{2}	12.85	$[M-H]^{-}$	327.3	182
[$^{2}H_{6}$]-20-HETE	1.99×10^{2}	11.80	$[M-H]^{-}$	325.3	281
[$^{2}H_{7}$]-5-KETE	2.00×10^{2}	14.95	$[M-H]^{-}$	324.2	210
[$^{2}H_{11}$]-11(12)-EpETrE	1.99×10^{2}	14.95	$[M-H]^{-}$	330.3	167
[$^{2}H_{11}$]-14.15-DiHETrE	2.00×10^{2}	10.40	$[M-H]^{-}$	348.3	207
[$^{2}H_{4}$]-9-HODE	2.00×10^{2}	12.70	$[M-H]^{-}$	299.3	172
[$^{2}H_{4}$]-9(10)-EpOME	2.00×10^{2}	14.55	$[M-H]^{-}$	299.1	172
[$^{2}H_{4}$]-9.10-DiHOME	2.01×10^{2}	10.17	$[M-H]^{-}$	317.2	203
SM 17:0	5.12×10^{3}	4.40	$[M+H]^{+}$	717.6	184.3
Cer 17:0	2.03×10^{3}	4.50	$[M+H-H2O]^{+}$	534.5	264.4
Cer[d17:1/24:1]	2.04×10^{3}	5.31	$[M+H-H2O]^{+}$	616.6	250.3
DhCer6:0	2.04×10^{3}	2.47	$[M+H]^{+}$	400.4	266.6
GlcCer 8:0	2.03×10^{3}	2.49	$[M+H-H2O]^{+}$	570.4	264.4
LacCer 17:0	2.04×10^{3}	4.14	$[M+H-H2O]^{+}$	858.8	264.4
[$^{2}H_{4}$]-AEA	5.69×10^{1}	5.33	$[M+H]^{+}$	352.2	66.0
[$^{2}H_{4}$]-PEA	5.27×10^{1}	6.21	$[M+H]^{+}$	304.2	66.0
[$^{2}H_{4}$]-LEA	3.66×10^{1}	5.39	$[M+H]^{+}$	328.2	66.0
[$^{2}H_{4}$]-D(h)EA	5.32×10^{1}	5.16	$[M+H]^{+}$	376.2	66.0
[$^{2}H_{4}$]-OEA	4.85×10^{1}	6.51	$[M+H]^{+}$	330.2	66.0
[$^{2}H_{5}$]-2-AG	2.08×10^{2}	5.85	$[M+H]^{+}$	384.3	269.1
