# Supplementary material for: Soluble epoxide hydrolase derived lipid mediators are elevated in bronchoalveolar lavage fluid from patients with sarcoidosis: a cross-sectional study
Source: Respir Res. 2018 Dec 3;19:236. doi: 10.1186/s12931-018-0939-0 (PMC6276236; doi:10.1186/s12931-018-0939-0)
Supplement: Supplementary file 4 — Appendix S1. Detailed procedures employed for the extraction of eicosanoids, endocannabinoids and sphingolipids. (PDF 11 kb) [file 12931_2018_939_MOESM4_ESM.pdf]

Appendix S1. Detailed procedures employed for the extraction of eicosanoids, endocannabinoids and sphingolipids.

Extraction of eicosanoids and endocannabinoids:

After activation of the cartridges with 2 mL of MeOH followed by 2 mL of water/methanol 95:5 with 0.1% acetic acid, the samples were adsorbed to the media by gravity, washed with 3 mL of water/methanol 95:5 with 0.1% acetic acid and dried in an extraction manifold under vacuum-induced air stream for 30 min. Lipids were eluted from the columns with 1 mL methanol and 2 mL of ethyl acetate in cryotubes containing 6 µL of glycerol in 30% methanol and dried on a Speedvac concentrator (Brand, Country). Samples were then reconstituted in 70 µl methanol, filtered using Amicon Ultrafree-MC, PVDF 0.1 µm filters (Millipore, US) at 4000 g for 3.5 minutes and transferred to LC-MS vials with 150 µl inserts for analysis.

Extraction of sphingolipids:

For the extraction of sphingolipids, 0.75 mL chloroform:methanol (1:2, v/v) were added and the samples were vortexed for 30 sec. Afterwards, 0.25 and 0.25 mL of methanol and water were sequentially added with 30 sec of vortex for each step. Samples were centrifuged for 5 min at 3000 *g* at 4°C and the lower organic phase was removed. The water phase was re-extracted by adding 0.3 mL of chloroform and centrifuged again for phase separation. The two organic phases were pooled, concentrated to dryness with vacuum centrifugation and reconstituted in 100 µL of methanol. The quantification of hexosylceramides (HexCer) was performed using glucosylceramides (GlcCer) as calibrators, but the method is unable to discriminate GlcCer from galactosylceramide (GalCer) as they share identical mass transitions and retention times. Samples were finally resuspended in 100 µL methanol, filtered

using Amicon Ultrafree-MC, PVDF 0.1 µm filters at 4000 g for 3.5 minutes and transferred to LC-MS vials with 150 µl inserts for analysis.
