# Supplementary material for: Soluble epoxide hydrolase derived lipid mediators are elevated in bronchoalveolar lavage fluid from patients with sarcoidosis: a cross-sectional study
Source: Respir Res. 2018 Dec 3;19:236. doi: 10.1186/s12931-018-0939-0 (PMC6276236; doi:10.1186/s12931-018-0939-0)
Supplement: Supplementary file 5 — Appendix S2. Chromatographic and general MS conditions employed in the detection of eicosanoids, sphingolipids and endocannabinoids. (PDF 97 kb) [file 12931_2018_939_MOESM5_ESM.pdf]

Appendix S2. Chromatographic and general MS conditions employed in the detection of eicosanoids, sphingolipids and endocannabinoids.

LC-MS/MS Eicosanoids:

Column: ACQUITY UPLC BEH (Ethylene Bridged Hybrid) C18 Column. 130Å. 1.7 µm. 2.1 mm X 150 mm (Product Number: 186002353) equipped with a pre-column (ACQUITY UPLC BEH C18 VanGuard Pre-column. 130Å. 1.7 µm. 2.1 mm X 5 mm. Product Number: 186003975). Both from Waters Corp. General chromatographic parameters: mobile phase A = 0.1 % acetic acid in water; mobile phase B = Acetonitrile / Isopropanol (90:10. v:v); with a flowrate of 500 µl/min and a column temperature 60°C. The chromatographic gradient was as follows: 0 min 10 % B; 0 → 3.5 min. 10 → 35 % B; 3.5 → 5.5 min. 35 → 40 % B; 5.5 → 7.0 min. 40 % B → 42 % B; 7.0 min → 9.0 min. 42 → 50 % B; 9 → 15 min. 50 → 65 % B; 15 → 16.0 min. 65 → 72.5 % B; 16.0 → 17 min. 72.5 → 95 % B; 17 → 18 min 95 % B (isocratic); 18 → 18 min. 95 → 10 % B; 18 → 21 min 10 % B (isocratic column conditioning). The general MS parameters were set as follows: Desolvation temperature: 600 ° C; Capillary Voltage: -2 kV; Desolvation gas (L/hr): 1000; Polarity mode: Negative. Compounds were quantified using standard curves and ratios of the peak area of each compound to IS. Calibration curves were calculated by least-squares linear regression with 1/x weighting. Further details on the method including MS transitions have been detailed elsewhere [1]. For samples where less than 3 mL were extracted (n=4), concentrations were normalized to the extracted volume.

LC-MS/MS Sphingolipids:

Column: ACQUITY UPLC BEH (Ethylene Bridged Hybrid) C8 Column. 130Å. 1.7 µm. 2.1 mm X 150 mm (Product Number: 186003377) equipped with a pre-column (ACQUITY UPLC BEH C8 VanGuard Pre-column. 130Å. 1.7 µm. 2.1 mm X 5 mm. Product Number: 186003978). both from Waters Corp. General chromatographic parameters: mobile phase A = 5mM ammonium formate / 0.2 % formic acid in water; mobile phase B = 5mM ammonium formate / 0.2 % formic acid in MeOH; with a flowrate of 375 µl/min and a column temperature 50°C. The chromatographic gradient was as follows: 0 min. 85% B; 0 → 4.5 min. 85% B; 4.5 → 8 min. 85 → 100% B; 8 → 8.5 min. 100% B; 8.5 → 9 min. 100 → 85% B; 9 → 11 min. 85% B (isocratic column conditioning). The general MS parameters were set as follows: Desolvation temperature: 550 ˚C; Capillary Voltage: 3 kV; Desolvation gas (L/hr): 800; Polarity mode: Positive. Compounds were quantified using standard curves and ratios of the peak area of SLs to IS. Calibration curves were calculated by least-squares linear regression with 1/x weighting. Further details on the method including MS transitions have been detailed elsewhere [2]. For samples where less than 3 mL were extracted (n=4), concentrations were normalized to the extracted volume.

LC-MS/MS Endocannibinoids:

Column: ACQUITY UPLC BEH (Ethylene Bridged Hybrid) C18 Column. 130Å. 1.7 µm. 2.1 mm X 150 mm (Product Number: 186002353) equipped with a pre-column (ACQUITY UPLC BEH C18 VanGuard Pre-column. 130Å. 1.7 µm. 2.1 mm X 5 mm. Product Number: 186003975). both from Waters Corp. General chromatographic parameters: mobile phase A = 0.1 % acetic acid in water; mobile phase B = Acetonitrile / Isopropanol (90:10. v:v); with a flowrate of 300 µl/min and a column temperature 50°C. The chromatographic gradient was as follows: 0 min. 65% B; 0 → 3.0 min. 65% B; 3.0 → 3.1 min. 65 → 80% B; 3.1 → 5.0 min.

80% B; 5.0 → 5.1 min. 80 → 90% B; 5.1 → 6.0 min. 90% B; 6.0 → 6.1 min. 90 → 100% B; 6.1 → 9.0 min. 100% B; 9.0 → 9.1 min. 100 → 65% B; 9.1 → 11.0 min. 65% B (isocratic column conditioning). The general MS parameters were set as follows: Desolvation temperature: 550 ° C; Capillary Voltage: 3 kV; Desolvation gas (L/hr): 800; Polarity mode: Positive. Compounds were quantified using standard curves and ratios of the peak area of each endocannabinoid to IS. Calibration curves were calculated by least-squares linear regression with 1/x weighting. Further details on the method including MS transitions have been detailed elsewhere [3].

Supplemental e-references

[1] Balgoma D, Yang M, Sjodin M, et al. Linoleic acid-derived lipid mediators increase in a female-dominated subphenotype of COPD. Eur Respir J. 2016;47(6):1645-1656.

[2] Checa A, Khademi M, Sar DG, et al. Hexosylceramides as intrathecal markers of worsening disability in multiple sclerosis. *Mult Scler.* 2015;21(10):1271-1279.

[3] Checa A, Holm T, Sjodin MO, et al. Lipid mediator profile in vernix caseosa reflects skin barrier development. *Sci Rep.* 2015;5:15740.
