# Supplementary material for: Soluble epoxide hydrolase derived lipid mediators are elevated in bronchoalveolar lavage fluid from patients with sarcoidosis: a cross-sectional study
Source: Respir Res. 2018 Dec 3;19:236. doi: 10.1186/s12931-018-0939-0 (PMC6276236; doi:10.1186/s12931-018-0939-0)
Supplement: Supplementary file 6 — Table S4. Results for the comparisons of lipid mediator levels between healthy (n = 16) and sarcoidosis (n = 41) groups. Values have been normalized to the median value of the healthy group. Compounds with p < 0.05 are highlighted in bold. (PDF 71 kb) [file 12931_2018_939_MOESM6_ESM.pdf]

Table S3. Results for the comparisons of lipid mediator levels between healthy (n=16) and sarcoidosis (n=41) groups. Values have been normalized to the median value of the healthy group. Compounds with $p < 0.05$ are highlighted in bold.

LIPID	Median control [Q1-Q3]	Median sarcoidosis [Q1-Q3]	p-val[a]	q-val[b]	Median FC[c]
PGD2	1.0 [0.8-1.7]	1.5 [0.5-3.7]	3.56×10^{-01}	2.79×10^{-01}	1.5
LTB4	1.0 [0.6-1.7]	0.7 [0.5-1.7]	2.78×10^{-01}	2.75×10^{-01}	0.7
5-HETE	1.0 [0.7-2.9]	0.8 [0.6-1.9]	1.95×10^{-01}	2.21×10^{-01}	0.8
9-HETE	1.0 [0.6-1.3]	1.6 [0.9-2.4]	2.01×10^{-01}	2.21×10^{-01}	1.6
11-HETE	1.0 [0.3-1.6]	1.0 [0.1-3.0]	8.59×10^{-01}	5.10×10^{-01}	1.0
15-HETE	1.0 [0.7-2.0]	2.0 [1.1-3.1]	1.02×10^{-01}	1.71×10^{-01}	2.0
15-KETE	1.0 [0.7-1.5]	1.1 [0.7-2.1]	5.76×10^{-01}	3.84×10^{-01}	1.1
5(6)-EpETrE	1.0 [0.5-1.2]	0.9 [0.6-1.2]	8.24×10^{-01}	5.00×10^{-01}	0.9
11(12)-EpETrE	1.0 [0.7-1.4]	1.1 [0.6-1.5]	7.15×10^{-01}	4.55×10^{-01}	1.1
**11,12-DiHETrE**	1.0 [0.2-3.2]	6.0 [3.0-10.0]	**4.44×10^{-05}**	**1.18×10^{-03}**	**6.0**
**14,15-DiHETrE**	1.0 [0.7-1.5]	1.8 [1.1-2.4]	**3.56×10^{-03}**	**3.17×10^{-02}**	**1.8**
9-HODE	1.0 [0.7-1.5]	1.3 [0.7-3.0]	3.28×10^{-01}	2.79×10^{-01}	1.3
**13-HODE**	1.0 [0.8-1.7]	1.6 [1.2-2.7]	**3.28×10^{-02}**	**1.31×10^{-01}**	**1.6**
13-KODE	1.0 [0.7-1.3]	1.4 [0.7-2.2]	3.55×10^{-01}	2.79×10^{-01}	1.4
EKODE	1.0 [0.6-1.6]	1.1 [0.5-2.1]	5.56×10^{-01}	3.80×10^{-01}	1.1
**9(10)-EpOME**	1.0 [0.7-1.2]	0.6 [0.4-0.8]	**3.94×10^{-02}**	**1.31×10^{-01}**	**0.6**
**12(13)-EpOME**	1.0 [0.7-1.2]	0.7 [0.4-0.9]	**4.49×10^{-02}**	**1.33×10^{-01}**	**0.7**
9,10-DiHOME	1.0 [0.5-1.6]	0.9 [0.6-1.2]	6.63×10^{-01}	4.32×10^{-01}	0.9
12,13-DiHOME	1.0 [0.5-1.6]	0.7 [0.4-1.1]	4.40×10^{-01}	3.26×10^{-01}	0.7
12(13)-EpODE	1.0 [0.5-1.7]	0.8 [0.4-1.1]	3.33×10^{-01}	2.79×10^{-01}	0.8
5-HETrE	1.0 [0.6-2.2]	0.7 [0.3-1.7]	2.27×10^{-01}	2.33×10^{-01}	0.7
15-HETrE	1.0 [0.5-1.9]	1.9 [1.0-2.8]	6.22×10^{-02}	1.38×10^{-01}	1.9
13-HOTrE	1.0 [0.7-1.8]	1.7 [0.9-2.9]	1.83×10^{-01}	2.21×10^{-01}	1.7
5-HEPE	1.0 [0.5-2.8]	0.6 [0.4-1.1]	1.89×10^{-01}	2.21×10^{-01}	0.6
14-HDoHE	1.0 [0.4-2.0]	1.9 [0.8-2.4]	1.86×10^{-01}	2.21×10^{-01}	1.9
17-HDoHE	1.0 [0.5-1.9]	1.6 [0.9-2.4]	8.66×10^{-02}	1.63×10^{-01}	1.6
AG	1.0 [0.7-1.3]	1.2 [0.9-1.5]	2.07×10^{-01}	2.21×10^{-01}	1.2
LEA	1.0 [1.0-1.3]	1.0 [1.0-2.0]	3.21×10^{-01}	2.79×10^{-01}	1.0
PEA	1.0 [0.5-33.8]	11.0 [0.6-23]	5.11×10^{-01}	3.69×10^{-01}	11.0
LG	1.0 [0.8-1.4]	1.4 [0.9-1.7]	1.83×10^{-01}	2.21×10^{-01}	1.4
SM 12:0	1.0 [0.5-1.6]	1.0 [0.0-1.5]	8.15×10^{-01}	5.00×10^{-01}	1.0
**SM 16:0**	1.0 [1.0-1.2]	1.3 [1.1-1.8]	**1.31×10^{-03}**	**1.74×10^{-02}**	**1.3**

**SM 18:0**	1.0 [0.9-1.1]	1.2 [0.9-1.6]	**2.70×10^{-02}**	**1.31×10^{-01}**	**1.2**
SM 18:1	1.0 [0.5-1.4]	1.5 [0.5-2.5]	1.82×10^{-01}	2.21×10^{-01}	1.5
**SM 24:1**	1.0 [0.7-1.4]	1.5 [0.9-2.0]	**3.61×10^{-02}**	**1.31×10^{-01}**	**1.5**
SM 24:0	1.0 [0.8-1.2]	0.9 [0.7-1.6]	9.72×10^{-01}	5.64×10^{-01}	0.9
Cer 16:0	1.0 [0.8-1.4]	1.4 [0.9-2.0]	9.15×10^{-02}	1.63×10^{-01}	1.4
Cer 18:0	1.0 [0.6-1.3]	1.3 [0.7-1.7]	5.73×10^{-02}	1.38×10^{-01}	1.3
Cer 20:0	1.0 [0.9-1.4]	1.4 [1.0-1.9]	6.77×10^{-02}	1.39×10^{-01}	1.4
Cer 22:0	1.0 [0.8-1.2]	1.1 [0.8-1.4]	5.51×10^{-01}	3.80×10^{-01}	1.1
Cer 24:1	1.0 [0.9-1.3]	1.3 [1.0-1.7]	5.74×10^{-02}	1.38×10^{-01}	1.3
Cer 24:0	1.0 [0.8-1.1]	1.1 [0.9-1.3]	4.39×10^{-01}	3.26×10^{-01}	1.1
**HexCer 16:0**	1.0 [1.0-1.3]	1.6 [1.1-2.3]	**1.56×10^{-02}**	**1.04×10^{-01}**	**1.6**
HexCer 18:0	1.0 [0.9-1.2]	1.2 [0.8-1.8]	1.18×10^{-01}	1.86×10^{-01}	1.2
HexCer 24:1	1.0 [0.8-1.2]	1.2 [0.9-1.4]	2.97×10^{-01}	2.79×10^{-01}	1.2
LacCer 16:0	1.0 [0.7-2.4]	1.4 [0.9-2.4]	3.45×10^{-01}	2.79×10^{-01}	1.4
LacCer 24:1	1.0 [0.6-1.5]	0.9 [0.6-1.5]	9.93×10^{-01}	5.64×10^{-01}	0.9

[a] Mann-Whitney p-value (two-tailed)

[b] Storey q-value

[c] Median sarcoidosis / Median healthy

Q1: Quartile 1

Q3: Quartile 3
