# Supplementary material for: Soluble epoxide hydrolase derived lipid mediators are elevated in bronchoalveolar lavage fluid from patients with sarcoidosis: a cross-sectional study
Source: Respir Res. 2018 Dec 3;19:236. doi: 10.1186/s12931-018-0939-0 (PMC6276236; doi:10.1186/s12931-018-0939-0)
Supplement: Supplementary file 7 — Table S5. Spearman’s correlations between lipid mediators and age for healthy controls and patients with sarcoidosis. Compounds with p < 0.05 are highlighted in bold. (PDF 39 kb) [file 12931_2018_939_MOESM7_ESM.pdf]

Table S4. Spearman´s correlations between lipid mediators and age for healthy controls and patients with sarcoidosis. Compounds with $p < 0.05$ are highlighted in bold. None of the comparisons presented a $q < 0.05$.

Lipid Mediator	Healthy		Sarcoidosis	
	r_s	p-val	r_s	p-val
PGD2	0.17	5.26×10^{-01}	0.06	7.03×10^{-01}
LTB4	-0.20	4.47×10^{-01}	-0.28	7.57×10^{-02}
5-HETE	-0.30	2.66×10^{-01}	-0.27	8.74×10^{-02}
9-HETE	0.03	9.04×10^{-01}	-0.13	4.07×10^{-01}
11-HETE	0.40	1.24×10^{-01}	-0.17	2.91×10^{-01}
15-HETE	-0.09	7.37×10^{-01}	-0.09	5.89×10^{-01}
15-KETE	0.30	2.51×10^{-01}	-0.11	4.94×10^{-01}
5(6)-EpETrE	-0.30	2.62×10^{-01}	-0.13	4.05×10^{-01}
11(12)-EpETrE	-0.37	1.58×10^{-01}	0.10	5.39×10^{-01}
11,12-DiHETrE	0.27	3.05×10^{-01}	0.18	2.71×10^{-01}
14,15-DiHETrE	-0.35	1.87×10^{-01}	0.00	9.76×10^{-01}
9-HODE	0.36	1.69×10^{-01}	-0.12	4.48×10^{-01}
13-HODE	0.38	1.44×10^{-01}	-0.01	9.31×10^{-01}
**13-KODE**	**0.52**	$\mathbf{4.04 \times 10^{-02}}$	-0.16	3.30×10^{-01}
EKODE	0.47	6.78×10^{-02}	-0.18	2.70×10^{-01}
9(10)-EpOME	-0.37	1.61×10^{-01}	0.26	1.07×10^{-01}
12(13)-EpOME	-0.31	2.37×10^{-01}	0.30	6.08×10^{-02}
9,10-DiHOME	0.08	7.58×10^{-01}	0.21	1.89×10^{-01}
12,13-DiHOME	0.09	7.45×10^{-01}	0.16	3.16×10^{-01}
12(13)-EpODE	0.04	8.86×10^{-01}	0.28	7.10×10^{-02}
5-HETrE	-0.13	6.35×10^{-01}	-0.23	1.52×10^{-01}
15-HETrE	0.00	9.98×10^{-01}	-0.09	5.85×10^{-01}
13-HOTrE	-0.12	6.59×10^{-01}	0.02	8.95×10^{-01}
5-HEPE	-0.25	3.57×10^{-01}	-0.12	4.58×10^{-01}
14-HDoHE	-0.20	4.63×10^{-01}	0.16	3.17×10^{-01}
17-HDoHE	-0.04	8.86×10^{-01}	0.10	5.42×10^{-01}
AG	-0.28	2.88×10^{-01}	-0.05	7.67×10^{-01}
**LEA**	-0.38	1.44×10^{-01}	**0.32**	$\mathbf{4.22 \times 10^{-02}}$
**PEA**	0.04	8.69×10^{-01}	0.32	$\mathbf{4.01 \times 10^{-02}}$
LG	-0.27	3.03×10^{-01}	0.02	8.90×10^{-01}
SM 12:0	0.09	7.29×10^{-01}	-0.05	7.50×10^{-01}
SM 16:0	0.17	5.41×10^{-01}	-0.18	2.57×10^{-01}
SM 18:0	-0.22	4.17×10^{-01}	-0.08	6.39×10^{-01}
SM 18:1	-0.36	1.69×10^{-01}	-0.12	4.41×10^{-01}
SM 24:1	0.02	9.50×10^{-01}	-0.19	2.43×10^{-01}

SM 24:0	0.19	4.88×10^{-01}	-0.04	7.95×10^{-01}
Cer 16:0	0.01	9.80×10^{-01}	0.30	5.91×10^{-02}
Cer 18:0	0.09	7.26×10^{-01}	0.26	1.02×10^{-01}
Cer 20:0	0.28	2.93×10^{-01}	0.12	4.48×10^{-01}
Cer 22:0	0.25	3.54×10^{-01}	0.21	1.87×10^{-01}
Cer 24:1	0.06	8.30×10^{-01}	0.04	7.92×10^{-01}
Cer 24:0	-0.01	9.71×10^{-01}	0.06	7.13×10^{-01}
HexCer 16:0	-0.14	6.01×10^{-01}	0.18	2.62×10^{-01}
HexCer 18:0	0.14	5.99×10^{-01}	0.22	1.65×10^{-01}
HexCer 24:1	0.12	6.52×10^{-01}	0.09	5.94×10^{-01}
**LacCer 16:0**	-0.24	3.76×10^{-01}	0.31	**4.64×10^{-02}**
**LacCer 24:1**	-0.24	3.75×10^{-01}	0.34	**2.91×10^{-02}**
