# Supplementary material for: Soluble epoxide hydrolase derived lipid mediators are elevated in bronchoalveolar lavage fluid from patients with sarcoidosis: a cross-sectional study
Source: Respir Res. 2018 Dec 3;19:236. doi: 10.1186/s12931-018-0939-0 (PMC6276236; doi:10.1186/s12931-018-0939-0)
Supplement: Supplementary file 8 — Table S6. Results of the Cochran-Armitage test with radiography stage in sphingolipids significantly differing (p < 0.05) between patients and controls. Direction of the null hypothesis is indicated for each compound. Compounds with p < 0.05 are highlighted in bold. (PDF 79 kb) [file 12931_2018_939_MOESM8_ESM.pdf]

**Table S5.** Results of the Cochran-Armitage test with radiography stage in sphingolipids significantly differing (p<0.05) between patients and controls. Direction of the null hypothesis is indicated for each compound. Compounds with p < 0.05 are highlighted in bold.

| **LIPID** | **p-val** | **$H_0$** |
|---|---|---|
| **11,12-DiHETrE** | 1.48E-04 | ↑ |
| 14,15-DiHETrE | 7.46E-02 | ↑ |
| 13-HODE | 7.51E-02 | ↑ |
| **SM 16:0** | 1.38E-02 | ↑ |
| SM 18:0 | 3.43E-01 | ↑ |
| SM 24:1 | 1.40E-01 | ↑ |
| HexCer 16:0 | 4.13E-01 | ↑ |
| **9(10)-EpOME** | 3.40E-02 | ↓ |
| **12(13)-EpOME** | 3.40E-02 | ↓ |
